# Interpretable Machine Learning for Predicting Enrofloxacin Residues in Fish Using a Large Literature-Derived Database

**DOI:** 10.3390/foods15142522

**Published:** 2026-07-16

**Authors:** Peilong Song, Bo Rong, Linhua Zhou, Haizhou Jiang, Liping Qiu, Wenjun Che, Longxiang Fang, Shunlong Meng, Chao Song

**Affiliations:** 1 School of Marine Technology and Environment, Dalian Ocean University, Dalian 116023, China; 17639525067@163.com (P.S.); r2403432988@163.com (B.R.); 13840232412@163.com (L.Z.); 2 Freshwater Fisheries Research Center, Chinese Academy of Fishery Sciences, Wuxi 214081, China; jianghaizhou@ffrc.cn (H.J.); qiulp@ffrc.cn (L.Q.); fanglongxiang@ffrc.cn (L.F.); 3 Laboratory of Quality & Safety Risk Assessment for Aquatic Products on Environmental Factors (Wuxi), Ministry of Agriculture and Rural Affairs, Wuxi 214081, China; 4 Wuxi Fisheries College, Nanjing Agricultural University, Wuxi 214081, China; 5 Key Laboratory of Freshwater Fisheries and Germplasm Resources Utilization, Ministry of Agriculture and Rural Affairs, Freshwater Fisheries Research Center, Chinese Academy of Fishery Sciences, Wuxi 214081, China; 6 Changzhou Dixin Biotechnology Co., Ltd., Changzhou 213161, China; cwj1218@126.com

**Keywords:** enrofloxacin, fish, residue prediction, machine learning, literature-derived database, source-grouped validation

## Abstract

Enrofloxacin use in aquaculture can lead to residue accumulation in fish tissues, creating challenges for food safety control and residue risk assessment. Residue prediction across published studies remains difficult because concentrations are influenced by species, tissue matrix, administration conditions, environmental factors, and between-study heterogeneity. Here, we constructed a literature-derived database to model matrix-specific enrofloxacin concentrations in fish. After duplicate removal, fish-focused filtering, source cleaning, and exclusion of records with missing critical variables, 1254 records from 39 source groups with traceable literature identifiers were retained from 2275 extracted records. Eleven predictors were used, and eight machine learning models were trained using log1p-transformed concentrations. Under a random row-level 80:20 split, the histogram gradient-boosting decision tree achieved the best row-level performance, with R^2^_log = 0.8889 and root-mean-squared error in log1p space (RMSE_log) = 0.2728. In contrast, stratified source-grouped validation showed markedly reduced performance, indicating limited cross-study generalization to unseen source groups. ExtraTrees showed the most stable grouped validation performance and was used for model-based interpretation. Species, tissue or biological matrix, dosing frequency, and sampling time point were highly ranked model-associated predictors. This study provides a transparent literature integration framework for residue pattern modeling and predictor prioritization, while highlighting the need for standardized residue depletion data and external validation.

## 1. Introduction

Enrofloxacin is a broad-spectrum fluoroquinolone antibiotic used for the prevention and treatment of bacterial infections in aquaculture [1,2]. Following administration, enrofloxacin and its major metabolite ciprofloxacin may persist in fish plasma and tissues for extended periods, with depletion profiles varying by species, tissue or biological matrix, dosing regimen, and water temperature [2,3,4,5]. These residues are directly relevant to food safety control, withdrawal period determination, and regulatory compliance because fluoroquinolones have been detected in commercial or cultured fish products from different aquaculture and market settings [6,7]. Therefore, developing transparent prediction approaches and identifying predictors associated with residue variation are important for aquaculture residue risk assessment [8].

Conventional investigations of enrofloxacin residues have mainly relied on pharmacokinetic experiments, tissue residue assays, depletion studies, and empirical statistical analyses. Although these approaches provide valuable evidence under controlled conditions, they are often restricted to a single species, a specific dosing regimen, a limited set of tissues, or a narrow environmental setting, as illustrated by species-specific studies in Yellow River carp, crucian carp, yellow catfish, rainbow trout, and largemouth bass [2,3,4,5,9,10]. These restrictions make it difficult to derive generalized conclusions across studies or broader aquaculture scenarios. In practice, residue formation is rarely governed by a single variable. Rather, it emerges from the coupled effects of species, tissue type, body weight, health status, dose, administration route, dosing frequency, sampling time, formulation, and environmental conditions such as water temperature and pH. These variables jointly influence absorption, distribution, metabolism, and excretion, resulting in a system characterized by marked heterogeneity, nonlinearity, and multi-factor interactions. Compared with conventional linear or small-sample analytical approaches, machine learning offers a promising strategy for modeling such complex relationships because it can capture nonlinear structures in heterogeneous tabular data while accommodating both continuous and categorical predictors [11]. However, two challenges remain central for enrofloxacin residue prediction. First, residue concentrations typically exhibit a pronounced right-skewed long-tail distribution, making direct modeling on the original scale unstable and sensitive to rare extreme observations. Second, predictive accuracy alone is insufficient for practical decision making; it is equally important to identify which variables are associated with model predictions and how they shape prediction outcomes.

To address these issues, the present study constructed a literature-derived database for modeling matrix-specific enrofloxacin concentrations in fish. Published studies were systematically retrieved, screened, and converted into structured observation-level records. Experimental conditions, biological characteristics, administration-related variables, and residue outcomes were extracted and harmonized using Python-assisted summaries followed by manual verification. Based on the cleaned dataset, eight machine learning models were benchmarked under both random row-level validation and source-grouped validation. Model-based interpretation was then conducted using feature importance, permutation importance, Shapley additive explanations (SHAP), partial dependence plots (PDP), individual conditional expectation (ICE), and residual diagnostics. The aim of this study was to provide a transparent literature integration and machine learning framework for residue pattern modeling and biologically plausible predictor prioritization, while carefully distinguishing within-database prediction from cross-study generalization.

## 2. Materials and Methods

### 2.1. Database Construction

**Figure 1:**
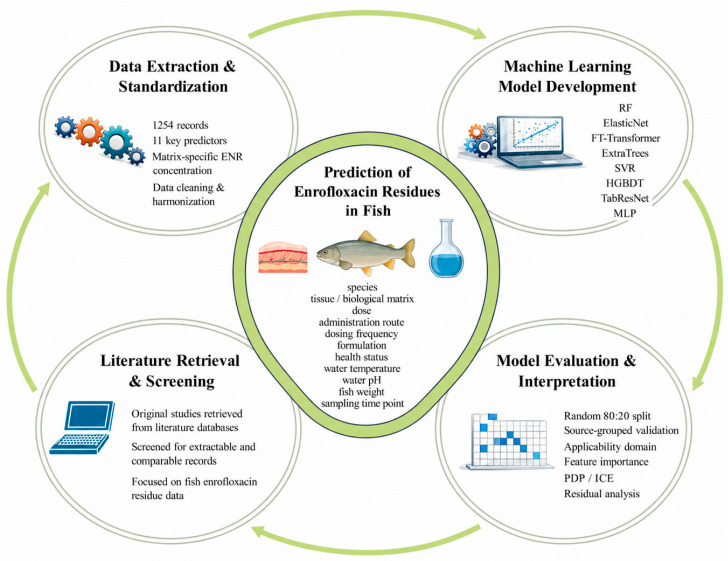
Overall workflow of the present study for predicting enrofloxacin residues in fish. The framework includes literature retrieval and screening, raw data extraction, duplicate removal, fish-focused scope filtering, variable harmonization, model development, random row-level validation, source-grouped validation, and model-based interpretation. A raw literature-derived database containing 2275 extracted records was first established. After data auditing and cleaning, the final main modeling dataset contained 1254 records from 39 source groups with traceable literature identifiers and was used for model training, evaluation, and interpretation.

The overall workflow of the present study, including literature retrieval, data extraction, data cleaning, model development, source-grouped validation, and model-based interpretation, is summarized in Figure 1. To construct a literature-derived database for enrofloxacin residue prediction in fish, original studies were retrieved from Web of Science, PubMed, Scopus, CNKI, and Google Scholar. The literature search was conducted up to 20 May 2026. The search terms included combinations of “enrofloxacin”, “fish”, “aquaculture”, “residue”, “withdrawal”, “tissue residue”, “pharmacokinetics”, and “residue depletion”. The detailed search strategy is provided in Table S1.

Studies were included if they reported quantitative enrofloxacin concentration data in fish-related biological matrices and if the residue values could be linked to at least part of the following information: fish species, tissue or biological matrix, administration route, dose, sampling time point, water temperature, water pH, fish weight, formulation, dosing frequency, or health status. Studies were excluded from the main modeling dataset if they only reported environmental matrices such as water or sediment, non-fish aquatic animals, plant matrices, toxicity endpoints, method validation spiked samples, or results that could not be linked to a defined experimental unit.

A total of 1128 potentially relevant publications were initially identified. After title, abstract, and full-text screening, 321 eligible studies were retained for raw data extraction. From these studies, 2275 raw records were initially extracted. Exact duplicate records were then identified based on all extracted fields, and 643 duplicate records were flagged. After duplicate removal, fish-focused scope filtering, source cleaning, and exclusion of records with missing critical modeling variables, the final main modeling dataset contained 1254 records from 39 source groups with traceable literature identifiers. These records were used for subsequent model development and validation. The full inclusion and exclusion criteria, data-cleaning steps, and record retention summary are provided in Tables S2 and S3.

### 2.2. Data Extraction and Variable Harmonization

After literature screening, relevant experimental conditions and residue outcomes were extracted from eligible studies and converted into structured observational records. Each row was defined as an observation unit corresponding to a residue measurement record for a specific combination of literature source, fish species, administration route, dose, tissue or biological matrix, and sampling time point. In most cases, the record represented a reported mean concentration at the treatment-time-matrix level rather than an individual-fish measurement. This definition was used to clarify the unit of analysis and to account for the potential non-independence of records originating from the same study or residue depletion curve.

Only parent enrofloxacin was used as the response variable. Ciprofloxacin, the major metabolite of enrofloxacin, was not merged with the enrofloxacin target variable and was not used as a predictor because metabolite reporting was inconsistent across the collected studies. Enrofloxacin concentrations were harmonized as matrix-specific concentrations. Concentrations in solid fish tissues were expressed as μg g^−1^ wet weight, which is numerically equivalent to mg kg^−1^ wet weight, whereas concentrations in plasma, serum, and other fluid matrices were expressed as μg mL^−1^. Tissue or biological matrix type was retained as a categorical predictor to account for matrix-specific concentration scales and residue distribution patterns.

To facilitate cross-study integration, Python was used as an auxiliary tool to summarize variable names, field meanings, synonymous expressions, and reporting patterns across studies. These Python-assisted outputs were then manually checked against the original publications, and the final field definitions were determined according to author-verified criteria rather than automatic output alone. Uncertain or ambiguous records were discussed among the authors and were either corrected according to the source text or excluded from the main modeling dataset. No literature identifier was used as a model predictor. Literature ID and Source_Group were retained only for provenance tracking, data auditing, and grouped validation to avoid source-level information leakage.

The structured database was organized around 11 predictors required for enrofloxacin residue modeling: species, administration route, dose, tissue or biological matrix, sampling time point, water temperature, water pH, fish weight, health status, enrofloxacin formulation, and dosing frequency. Matrix-specific enrofloxacin concentration was used as the response variable. Additional information reported inconsistently across studies was retained only for record completion or contextual checking. After field harmonization and data auditing, the final main modeling dataset contained 1254 records from 39 source groups with traceable literature identifiers.

### 2.3. Data Cleaning, Missing-Value Handling, and Unit Conversion

Because the extracted records originated from multiple studies with heterogeneous experimental designs, reporting formats, matrix types, and measurement units, the raw data could not be used directly for model development. Therefore, a Python-based preprocessing and auditing pipeline was developed in the present study to perform duplicate checking, scope filtering, source cleaning, variable harmonization, missing-value assessment, unit standardization, and preparation of model-ready data.

Exact duplicate records were first identified based on all extracted fields. Records outside the scope of fish-related biological matrices, including environmental matrices, non-fish aquatic animals, plant matrices, toxicity endpoints, and method validation spiked samples, were excluded from the main modeling dataset. Records with missing source information, missing response values, missing sampling time points, or missing/non-positive dose values were also excluded, because these variables were required to define the observation unit or the response variable. A summary of the data-cleaning and record retention process is provided in Table S3.

All data-cleaning and modeling procedures were implemented in Python 3.10.2. The main packages included pandas 2.2.3, NumPy 2.2.6, scikit-learn 1.7.1 [12], PyTorch 2.9.1 + cpu, matplotlib 3.10.5, and SHAP 0.49.1. The preprocessing code was organized to ensure that imputation, scaling, and encoding parameters were estimated only from the training data within each validation scheme, thereby reducing the risk of information leakage.

For predictor variables retained in the main modeling dataset, missing values were handled within the model-preprocessing pipeline rather than by using information from the full dataset. Missing values in continuous predictors were imputed using the median value estimated from the training set, and the same fitted imputer was then applied to the validation or test data. Missing, ambiguous, or inconsistently reported categorical entries were encoded as “Unknown”. This strategy was used to preserve usable records while explicitly retaining uncertainty caused by incomplete reporting. The missing-value proportion of each predictor is reported in Table S5.

Unit harmonization was conducted using predefined rules. Dose was expressed as mg kg^−1^ body weight, sampling time as hours, water temperature as °C, water pH as unitless pH, and fish weight as grams. Enrofloxacin concentrations were treated as matrix-specific concentrations: solid fish tissue concentrations were expressed as μg g^−1^ wet weight, which is numerically equivalent to mg kg^−1^ wet weight, whereas plasma, serum, and other fluid matrix concentrations were expressed as μg mL^−1^. The tissue or biological matrix variable was retained as a categorical predictor to account for matrix-specific concentration scales and residue distribution patterns. The unit harmonization rules, assumptions, and affected variables are summarized in Table S4.

### 2.4. Feature System Construction and Preprocessing

Based on the cleaned main modeling dataset, matrix-specific enrofloxacin concentration was defined as the response variable. Eleven predictors were used as model inputs: species, administration route, dose, tissue or biological matrix, sampling time point, water temperature, water pH, fish weight, health status, enrofloxacin formulation, and dosing frequency. These predictors covered biological characteristics, administration-related factors, and environmental conditions associated with enrofloxacin residue variation in fish.

The continuous predictors included dose, sampling time point, water temperature, water pH, and fish weight. Dose was expressed as mg kg^−1^ body weight, sampling time point as hours, water temperature as °C, water pH as a unitless pH value, and fish weight as grams. The categorical predictors included species, tissue or biological matrix, administration route, dosing frequency, formulation, and health status. Categorical variables were encoded using one-hot encoding with unknown categories ignored during validation or testing. Missing values in continuous predictors were imputed using the median value estimated from the training set, whereas missing or ambiguous categorical values were encoded as “Unknown”.

All preprocessing operations were implemented within the model-training pipeline to reduce the risk of data leakage. For each validation scheme, imputers, one-hot encoders, and scalers were fitted only on the training data and then applied to validation or test data. Continuous predictors were standardized after imputation to support scale-sensitive models, including ElasticNet, SVR, MLP, FT-Transformer, and TabResNet. The same model-ready feature structure was used for all models to ensure comparability across algorithms. Literature ID, Source_Group, Observation_Unit_ID, and Curve_ID were not included as predictors.

### 2.5. Model Development and Performance Evaluation

To systematically compare the suitability of different algorithms for predicting matrix-specific enrofloxacin concentrations in fish, eight regression models were developed and benchmarked: Random Forest (RF) [13], ElasticNet, multilayer perceptron (MLP), Feature Tokenizer Transformer (FT-Transformer), ExtraTrees [14], support vector regression (SVR), histogram gradient-boosting decision tree (HGBDT) [15], and TabResNet. These models were selected to cover linear regularized regression, kernel-based regression, ensemble-tree-based learning, and neural-network-based tabular learning approaches. The FT-Transformer and TabResNet architectures were included as comparative deep tabular models following recent work on neural models for tabular data [16].

All models were trained using log1p-transformed matrix-specific enrofloxacin concentrations as the response variable because the original response variable showed a pronounced right-skewed distribution. Model predictions were evaluated both in the log1p-transformed space and on the back-transformed original scale. The primary model comparison focused on R^2^_log, RMSE_log, and MAE_log, while original-scale R^2^, RMSE, and MAE were also reported as supplementary indicators of concentration-scale prediction error.

Two validation schemes were used. First, a random row-level 80:20 train–test split was applied using a fixed random seed (random state = 42). This validation scheme was used to evaluate row-level predictive performance within the integrated literature-derived dataset. Second, five-fold stratified source-grouped validation was conducted using Source_Group as the grouping variable. In this validation scheme, all records from the same source group were assigned to the same fold, thereby reducing the risk that non-independent records from the same study or residue depletion curve appeared in both training and test subsets. The target distribution was stratified based on binned log1p-transformed concentrations to reduce fold imbalance. This grouped validation was used to assess cross-study generalization to unseen source groups. A summary of the source-grouped validation setup and model performance is provided in Table S8.

For the deep-learning models, 20% of the training data was used as an internal validation subset for early stopping. In the source-grouped validation, this validation split was generated within the training sources and was not mixed with the held-out test sources. Early stopping was based on validation RMSE_log. Because the cleaned main modeling dataset was modest in size, FT-Transformer and TabResNet were included as comparative neural tabular models rather than as presumed superior architectures, and their potential risk of overfitting was considered during interpretation.

The RF model was implemented with 800 trees, bootstrap sampling, max_features = 1.0, and min_samples_leaf = 1. ExtraTrees was configured with 800 trees, max_features = 1.0, and min_samples_leaf = 1. ElasticNet used alpha = 1 × 10^−3^, l1_ratio = 0.3, and max_iter = 20,000. The MLP model adopted a three-layer architecture with 256, 128, and 64 hidden units, and early stopping was enabled. SVR was implemented with a radial basis function kernel, C = 20.0, epsilon = 0.05, and gamma = scale. HGBDT was trained with learning_rate = 0.05 and max_iter = 800.

The two neural tabular models were trained on standardized inputs using AdamW optimization. Common training settings included a batch size of 128, a maximum of 200 epochs, an initial learning rate of 2 × 10^−4^, weight decay of 1 × 10^−2^, and gradient clipping at 1.0. Early stopping was applied with a patience of 20 epochs. The FT-Transformer used d_token = 192, 4 encoder layers, 8 attention heads, a feed-forward dimension of 384, and dropout = 0.1. TabResNet used a hidden dimension of 512, 4 residual blocks, and dropout = 0.15. Hyperparameters were selected based on preliminary model runs and were kept fixed across validation schemes to ensure comparability among models.

### 2.6. Interpretability Analysis

To interpret model behavior in a cautious and biologically informed manner, ExtraTrees was selected as the main model for interpretability analysis. Although HGBDT achieved the best performance under the random row-level split, ExtraTrees showed comparable random split performance and the most stable performance under stratified source-grouped validation. Therefore, ExtraTrees was used as the primary model for model-based interpretation.

A multi-level interpretability analysis was conducted. First, impurity-based feature importance was calculated from the fitted ExtraTrees model to provide an initial global ranking of predictors. Second, permutation importance was further used to evaluate the decrease in predictive performance after each predictor was randomly permuted, thereby providing a complementary importance estimate less directly dependent on tree-splitting frequency. Third, SHAP was used to characterize the direction and magnitude of feature contributions at the individual-sample level [17]. Fourth, PDP and ICE analyses were used to describe model-predicted response patterns for key continuous predictors and to examine heterogeneity among observations [15,18]. Rug plots were added to the PDP/ICE panels to indicate the observed feature distributions and to avoid overinterpretation in sparse data regions.

These interpretability analyses were used to identify model-associated predictor rankings and biologically plausible response patterns rather than to infer causal mechanisms. Because the database was derived from multiple literature sources, feature importance results may partly reflect study-level structure, species-specific experimental designs, tissue selection, and reporting patterns. Therefore, the interpretation was framed as model-based and hypothesis generating.

## 3. Results and Discussion

### 3.1. Database Characteristics

Based on the cleaned main modeling dataset, 1254 records from 39 source groups with traceable literature identifiers were retained for model development and validation. The dataset contained 11 predictors and one response variable, namely, matrix-specific enrofloxacin concentration. It covered 13 standardized fish species or fish groups, multiple tissue or biological matrix categories, and several administration routes. The predictors included biological characteristics, administration-related variables, and environmental variables, thereby providing a heterogeneous but structured basis for residue prediction.

**Figure 2:**
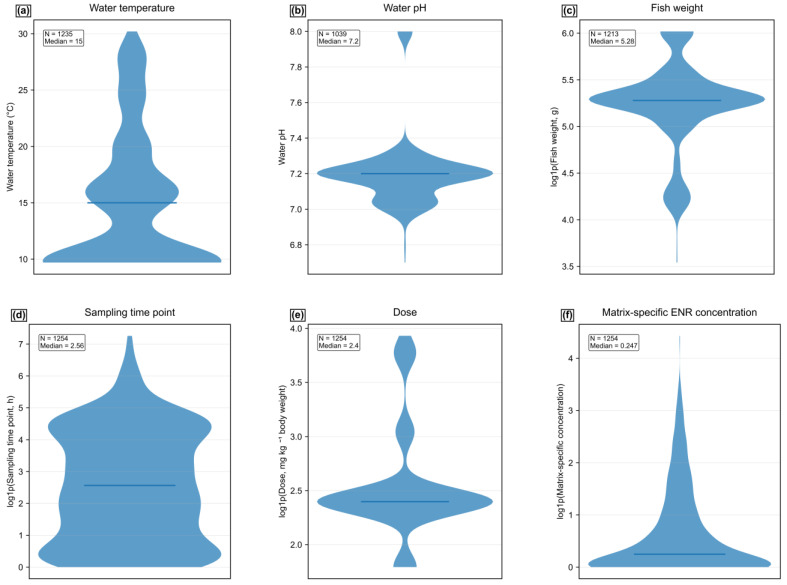
Violin plots of key continuous variables in the cleaned main modeling dataset: (**a**) water temperature, (**b**) water pH, (**c**) fish weight, (**d**) sampling time point, (**e**) dose, and (**f**) matrix-specific enrofloxacin concentration. Log1p transformation was applied to skewed variables to improve visual readability. The violin shape represents the probability density distribution, and the internal line indicates the median value.

**Figure 3:**
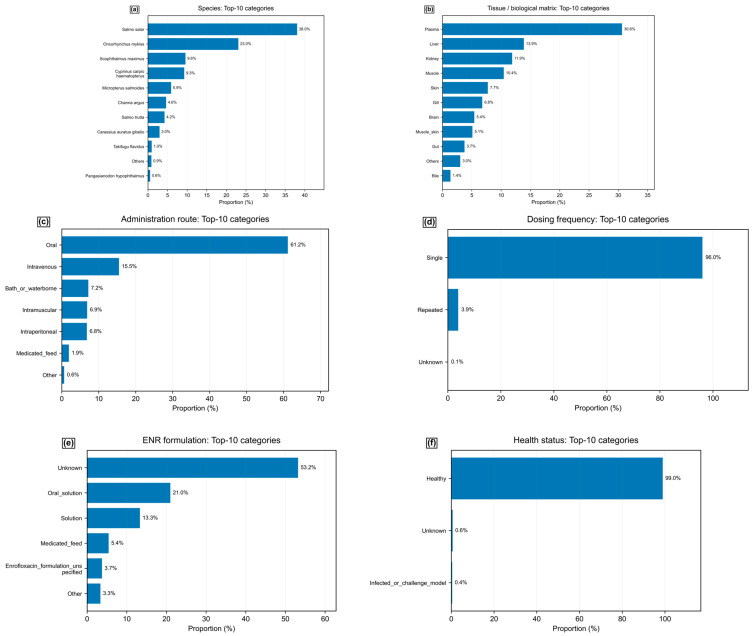
Top-10 composition proportions of categorical predictors in the cleaned main modeling dataset: (**a**) species, (**b**) tissue or biological matrix, (**c**) administration route, (**d**) dosing frequency, (**e**) enrofloxacin formulation, and (**f**) health status. For each categorical predictor, the ten most frequent categories are shown, and the remaining categories are grouped as “Others”.

**Figure 4:**
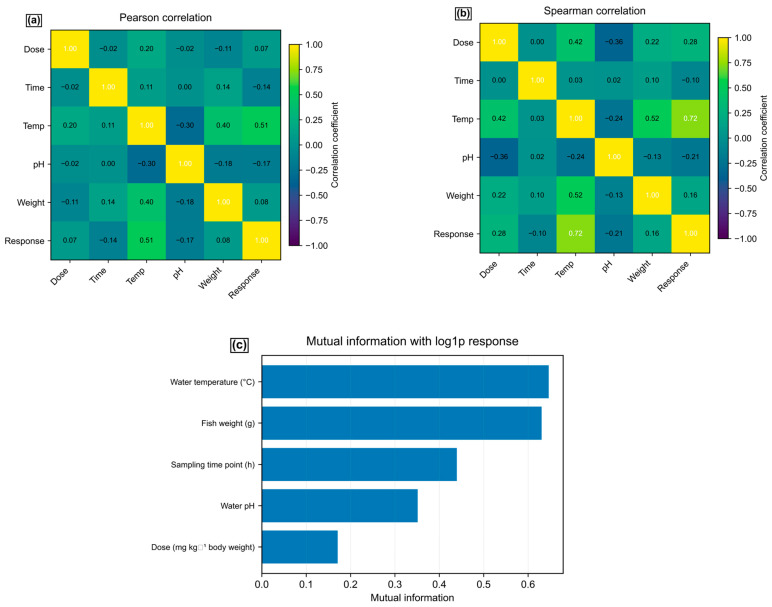
Correlation and nonlinear association analysis of continuous predictors and the response variable: (**a**) Pearson correlation, (**b**) Spearman correlation, and (**c**) mutual information with log1p-transformed matrix-specific enrofloxacin concentration.

Several statistical characteristics of the cleaned modeling dataset were noteworthy. First, matrix-specific enrofloxacin concentration showed a pronounced right-skewed long-tail distribution, indicating that a small number of high-concentration observations could exert a strong influence on model fitting on the original scale (Figure 2). Second, categorical predictors exhibited a head-heavy distribution, in which several common categories accounted for a large proportion of the data, whereas many categories were sparsely represented (Figure 3). This sparse tail structure suggested that model predictions for rare categories should be interpreted with greater caution. Third, Pearson and Spearman correlation analyses, together with mutual information analysis, showed that the relationships between individual continuous predictors and the response variable were generally weak to moderate (Figure 4). These results indicated that the prediction task was unlikely to be sufficiently described by isolated linear relationships alone and supported the use of nonlinear models capable of handling feature interactions in heterogeneous tabular data.

Descriptive statistics of the cleaned modeling dataset, including the median and range of matrix-specific enrofloxacin concentration, dose, sampling time point, water temperature, water pH, and fish weight, are provided in Table S6. Full category distributions for species, tissue or biological matrix, administration route, dosing frequency, formulation, and health status are provided in Table S7.

### 3.2. Predictive Performance of the Models

**Figure 5:**
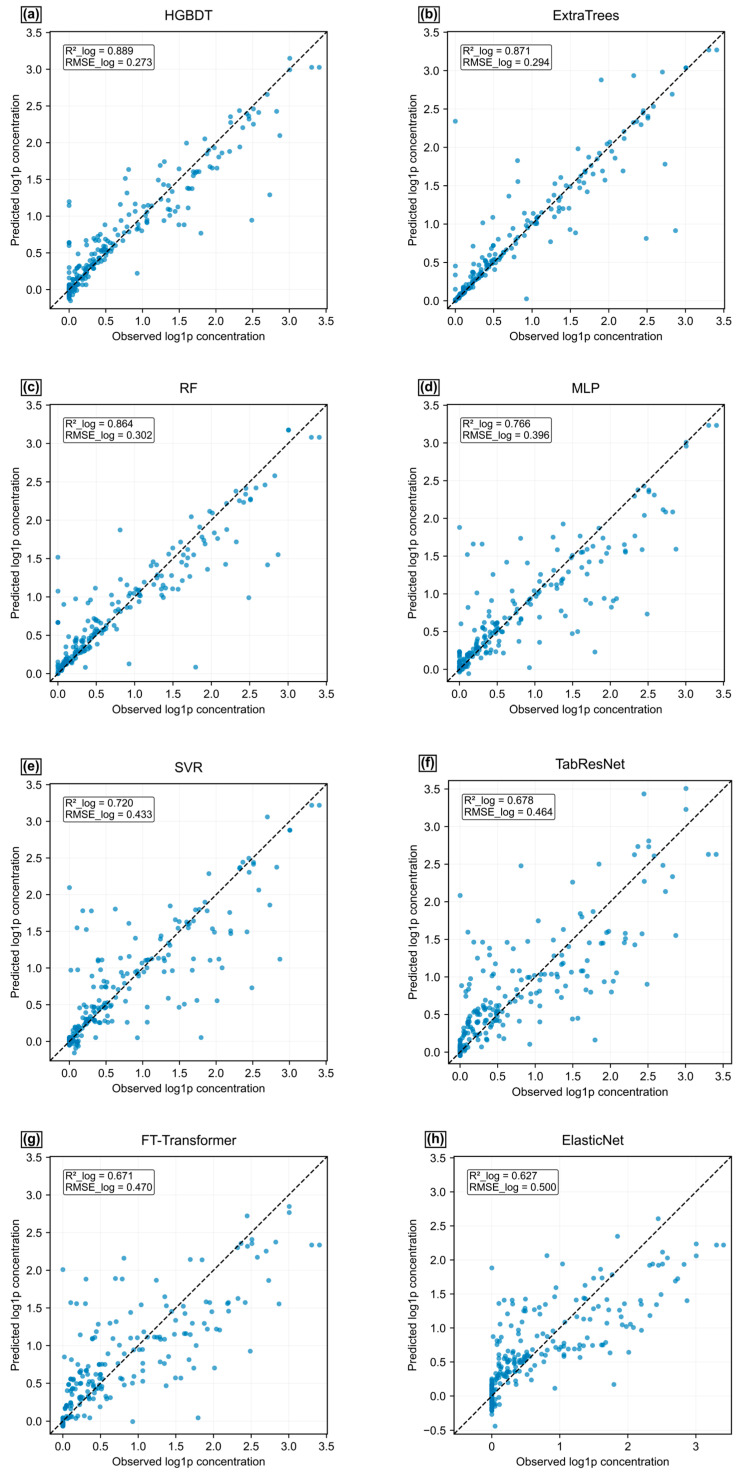
Observed versus predicted log1p-transformed matrix-specific enrofloxacin concentrations under the random row-level 80:20 split: (**a**) HGBDT, (**b**) ExtraTrees, (**c**) RF, (**d**) MLP, (**e**) SVR, (**f**) TabResNet, (**g**) FT-Transformer, and (**h**) ElasticNet. The dashed line represents the 1:1 line between observed and predicted values.

Under the random row-level 80:20 split, all eight models were trained and evaluated on the cleaned main modeling dataset (Table 1; Figure 5). Model performance was primarily assessed in the log1p-transformed space because the response variable showed a pronounced right-skewed distribution. In this validation scheme, HGBDT achieved the highest row-level predictive performance, with an R^2^_log of 0.8889 and an RMSE_log of 0.2728, followed by ExtraTrees (R^2^_log = 0.8714; RMSE_log = 0.2936) and RF (R^2^_log = 0.8641; RMSE_log = 0.3018). These results indicate that ensemble-tree-based models were effective at capturing residue patterns within the integrated literature-derived dataset.

However, because records from the same source group, experiment, or residue depletion curve may not be fully independent, random row-level performance should not be interpreted as direct evidence of cross-study generalization. Therefore, five-fold stratified source-grouped validation was further conducted using Source_Group as the grouping variable (Table 2). Under this stricter validation scheme, all models showed reduced performance compared with the random row-level split. ExtraTrees showed the most stable performance among the eight models, with an R^2^_log of −0.3409 ± 0.5023 and an RMSE_log of 0.7978. This decrease indicates that predicting residues for unseen source groups is substantially more challenging than row-level interpolation within the integrated database, likely because of between-study heterogeneity in species composition, dosing regimens, tissue or biological matrix type, analytical methods, and reporting practices.

Although HGBDT achieved the best performance under the random row-level split, ExtraTrees showed comparable random split performance and the most stable performance under stratified source-grouped validation. Therefore, ExtraTrees was retained as the main model for subsequent interpretability analysis. Overall, the model comparison suggests that random row-level validation reflects within-database interpolation ability, whereas source-grouped validation provides a more conservative estimate of cross-study generalization.

The applicability domain of the models was defined by the feature space represented in the cleaned training data. Predictions were considered more reliable for samples whose continuous predictors fell within the observed training ranges and whose categorical levels were sufficiently represented in the training set. In contrast, predictions for rare species, rare tissue or biological matrix categories, uncommon administration routes, extremely high residue concentrations, or feature combinations sparsely represented in the literature-derived dataset should be interpreted with caution. This limitation was further considered in the residual diagnostics and PDP/ICE analyses.

### 3.3. Key Influencing Factors

**Figure 6:**
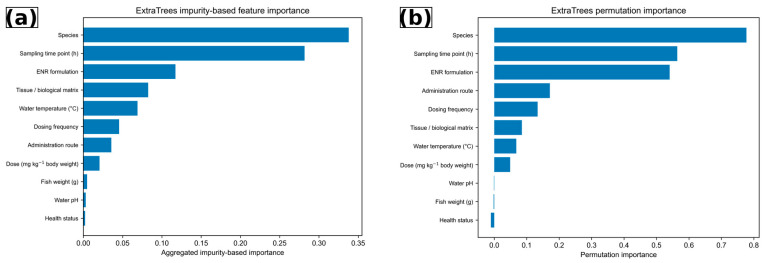
Global feature importance analysis of the ExtraTrees model: (**a**) aggregated impurity-based feature importance and (**b**) permutation importance. These results indicate model-associated predictor rankings rather than causal effects.

After model comparison, ExtraTrees was selected as the main model for interpretability analysis because it showed comparable random split performance and the most stable performance under stratified source-grouped validation. The feature-importance analysis showed that species was the highest-ranked model-associated predictor, accounting for approximately 32% of the model-derived feature importance (Figure 6). Tissue or biological matrix ranked second, contributing approximately 17%, followed by dosing frequency at approximately 15%. Together, these three predictors accounted for approximately 64% of the model-derived feature importance.

**Figure 7:**
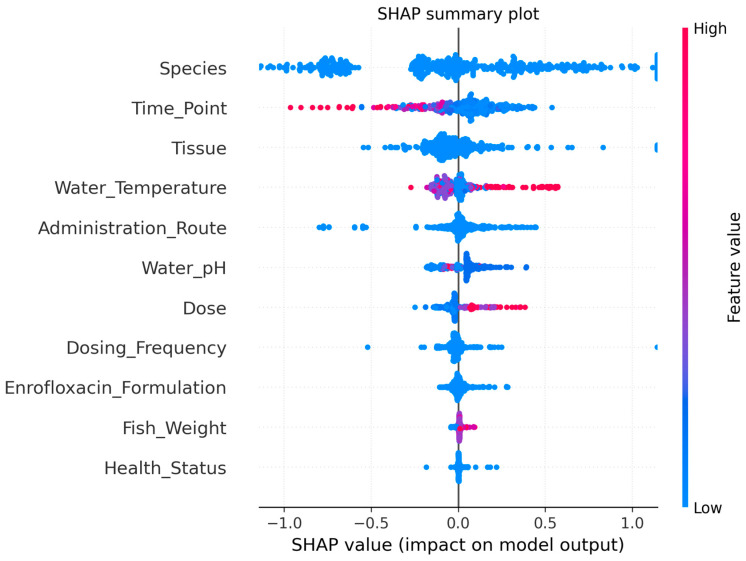
SHAP-based interpretation of the ExtraTrees model. SHAP values represent model-based feature contributions in the log1p-transformed matrix-specific enrofloxacin concentration space. These results indicate model-associated feature contribution patterns rather than causal effects.

The SHAP-based interpretation provided a complementary sample-level explanation of the ExtraTrees model and is shown in Figure 7.

These results suggest that model predictions were strongly associated with species-specific differences, matrix-specific residue distribution patterns, and dosing-related exposure information. The high ranking of species is biologically plausible because fish species may differ in absorption, distribution, metabolism, and excretion processes. However, this importance should not be interpreted as a direct measure of biological variance explained. Because the database was derived from multiple literature sources, the species effect may also partly reflect study-level structure, species-specific experimental designs, dosing protocols, sampled tissues, analytical methods, and reporting practices.

Tissue or biological matrix also showed a high model-derived importance, which is consistent with known differences in residue accumulation and depletion among edible tissues, metabolic organs, and fluid matrices. Dosing frequency and sampling time point were also important model-associated predictors, suggesting that the fitted model captured information related to cumulative exposure and post-administration depletion. Overall, the ranking pattern should be interpreted as a model-based prioritization of influential predictors rather than as evidence of causal residue determinants.

### 3.4. Nonlinear Effects, Heterogeneity, and Practical Implications

**Figure 8:**
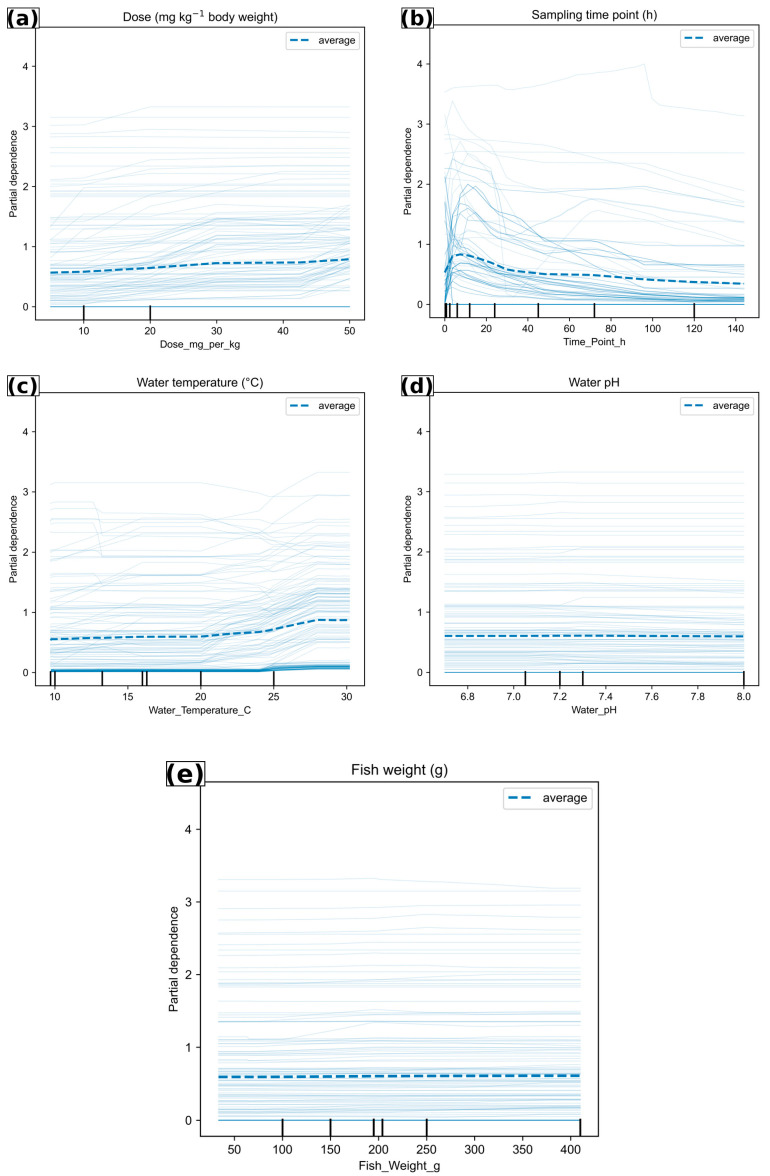
Partial dependence plots and individual conditional expectation curves for key continuous predictors in the ExtraTrees model: (**a**) dose, (**b**) sampling time point, (**c**) water temperature, (**d**) water pH, and (**e**) fish weight. The *y*-axis represents predicted log1p-transformed matrix-specific enrofloxacin concentration. The solid lines represent individual conditional expectation (ICE) curves for individual observations, while the dashed lines represent the average partial dependence (PDP) curves. Different shades of the colored areas indicate the variation among individual ICE curves. Rug plots indicate the observed distribution of each predictor. Rug plots indicate the observed distribution of each predictor.

PDP and ICE analyses were used to examine model-predicted response patterns for key continuous predictors, including dose, sampling time point, water temperature, water pH, and fish weight (Figure 8). The *y*-axis represents the predicted log1p-transformed matrix-specific enrofloxacin concentration. In the fitted ExtraTrees model, higher dose ranges were generally associated with higher predicted concentrations, whereas longer sampling time points were generally associated with lower predicted concentrations. These model response patterns are consistent with exposure accumulation and post-administration depletion, but they should not be interpreted as causal effects.

The ICE curves showed substantial heterogeneity among observations, indicating that the predicted response to a given continuous predictor varied across species, tissue or biological matrix types, dosing regimens, and literature sources. This heterogeneity helps explain why simple Pearson correlations were limited, while nonlinear models achieved better row-level predictive performance. However, PDP and ICE analyses can be influenced by feature correlations and may generate unrealistic feature combinations in sparse regions of the feature space. Therefore, rug plots were added to indicate the observed distribution of each predictor, and response patterns outside dense data regions were interpreted cautiously.

**Figure 9:**
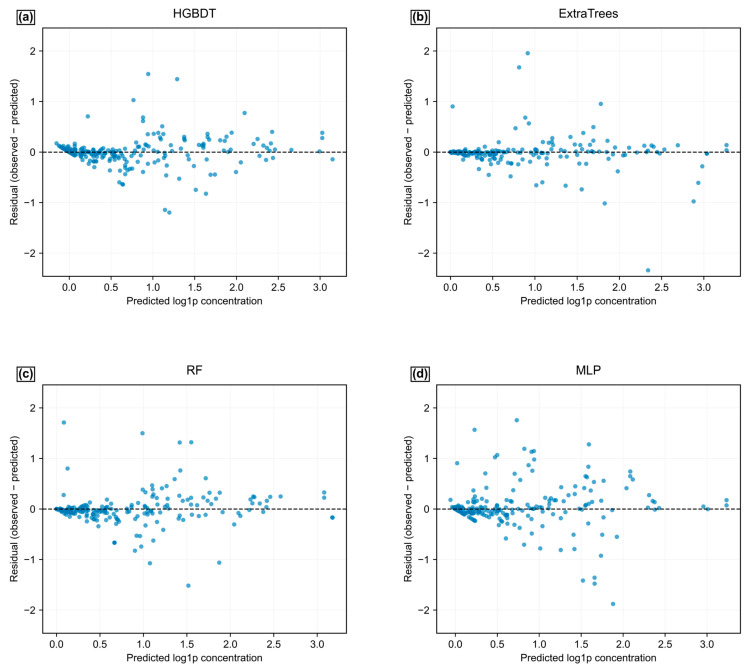

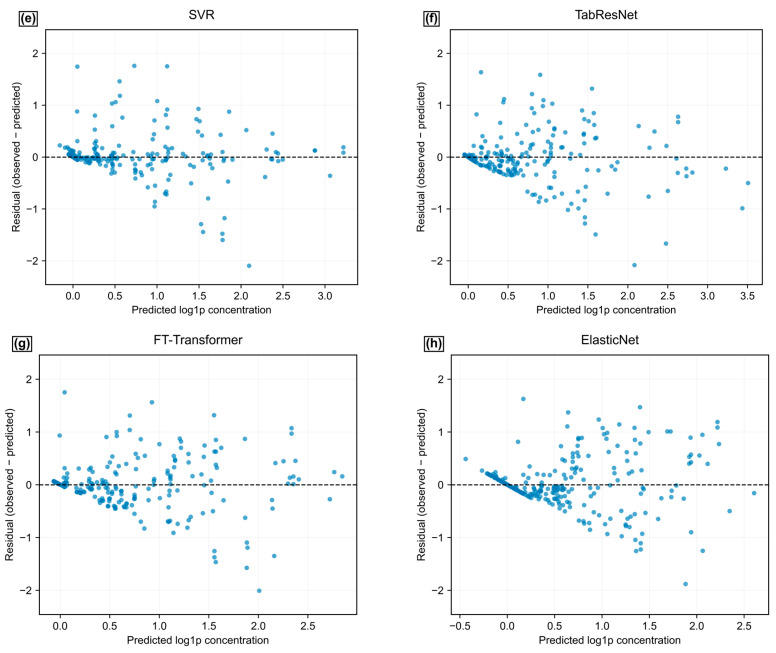
Residual diagnostics for the eight models under the random row-level 80:20 split: (**a**) HGBDT, (**b**) ExtraTrees, (**c**) RF, (**d**) MLP, (**e**) SVR, (**f**) TabResNet, (**g**) FT-Transformer, and (**h**) ElasticNet. Residuals were calculated in the log1p-transformed space as observed minus predicted values. The dashed line indicates the zero residual error reference line, where observed and predicted values are identical.

Residual diagnostics further showed that prediction errors were not evenly distributed across the response range (Figure 9). Although the random row-level split showed favorable overall performance, larger residuals were observed for some high-concentration samples and sparsely represented feature combinations. This pattern indicates that model reliability is higher within well-represented regions of the cleaned modeling dataset and lower for rare categories, extreme residue values, or unusual combinations of species, tissue or biological matrix, administration route, and sampling time point.

Collectively, the revised analysis provides a structured route from literature-derived data integration to residue pattern modeling and model-based predictor prioritization. The results suggest that the fitted models can capture important within-database residue patterns, especially under random row-level validation. However, the source-grouped validation results indicate that cross-study generalization remains challenging. Therefore, the present framework should be interpreted as a data-driven tool for literature integration, hypothesis generation, and model-associated predictor ranking, rather than as a direct substitute for external validation, regulatory residue assessment, or experimentally determined withdrawal-period studies.

## 4. Conclusions

Based on systematic literature retrieval, screening, structured data extraction, and data auditing, this study established a cleaned literature-derived dataset for modeling matrix-specific enrofloxacin concentrations in fish. From 2275 initially extracted records, 1254 records from 39 source groups with traceable literature identifiers were retained in the final main modeling dataset after duplicate removal, fish-focused scope filtering, source cleaning, and exclusion of records with missing critical modeling variables. The dataset included 11 predictors covering biological characteristics, administration-related factors, and environmental conditions.

The results showed that the response variable exhibited a pronounced right-skewed long-tail distribution, supporting the use of log1p transformation for model training and evaluation. Under the random row-level 80:20 split, HGBDT achieved the highest row-level predictive performance, followed by ExtraTrees and RF. However, stratified source-grouped validation showed markedly reduced performance for all models, indicating that cross-study generalization to unseen source groups was substantially more challenging than row-level interpolation within the integrated database. Among the evaluated models, ExtraTrees showed comparable random-split performance and the most stable source-grouped validation performance and was, therefore, retained as the main model for interpretability analysis.

Model-based interpretability analysis indicated that species, tissue or biological matrix, dosing frequency, and sampling time point were highly ranked model-associated predictors. These findings are biologically plausible because residue concentrations may vary with species-specific pharmacokinetic characteristics, matrix-specific distribution patterns, cumulative exposure, and post-administration depletion. However, these rankings should be interpreted as model-derived feature-importance patterns rather than causal evidence or direct estimates of biological variance explained, because study-level structure, sparse categories, and heterogeneous reporting practices may also influence the results.

Overall, this study provides a transparent literature integration and machine learning framework for residue pattern modeling and model-associated predictor prioritization. The framework may support hypothesis generation and help identify variables requiring closer attention in future residue studies. Future investigations should incorporate more standardized residue depletion experiments, harmonized reporting units, external validation datasets, and stricter leave-one-study-out or leave-one-species-out validation schemes. Combining literature-derived modeling with regulatory maximum residue limits and experimentally validated withdrawal period data may further improve the practical applicability of such models in aquaculture residue risk assessment.

## Figures and Tables

**Table 1:** Predictive performance of the eight models under the random row-level 80:20 split. Model performance was evaluated using matrix-specific enrofloxacin concentration as the response variable. R^2^, RMSE, and MAE were calculated on the back-transformed original scale, whereas R^2^_log, RMSE_log, and MAE_log were calculated in the log1p-transformed space.

Model	R^2^	RMSE	MAE	R^2^_log	RMSE_log	MAE_log
HGBDT	0.8480	1.6075	0.6218	0.8889	0.2728	0.1521
ExtraTrees	0.8045	1.8228	0.5487	0.8714	0.2936	0.1119
RF	0.8272	1.7137	0.6557	0.8641	0.3018	0.1488
MLP (sklearn)	0.7815	1.9270	0.8294	0.7665	0.3956	0.2103
SVR	0.7583	2.0267	0.8670	0.7198	0.4334	0.2272
TabResNet	0.5044	2.9023	1.2779	0.6781	0.4645	0.2933
FT-Transformer	0.5880	2.6462	1.1779	0.6706	0.4699	0.2965
ElasticNet	0.4508	3.0551	1.3868	0.6274	0.4997	0.3482

**Table 2:** Predictive performance of the eight models under five-fold stratified source-grouped validation. Source_Group was used as the grouping variable to ensure that all records from the same source group were assigned to the same fold. R^2^_log is reported as mean ± standard deviation across folds; RMSE_log is reported as the fold mean. A summary of the source-grouped validation setup and model performance is provided in Table S8.

Model	R^2^_log (Mean ± SD)	RMSE_log (Fold Mean)
ExtraTrees	−0.3409 ± 0.5023	0.7978
ElasticNet	−0.5261 ± 0.8683	0.8433
RF	−0.9608 ± 1.4324	0.9056
TabResNet	−1.3604 ± 1.8396	0.9676
SVR	−1.3912 ± 2.0492	0.9561
FT-Transformer	−1.5749 ± 2.4346	0.9760
MLP	−1.7552 ± 1.5110	1.1740
HGBDT	−1.8137 ± 2.6146	1.0138

## Data Availability

The cleaned main modeling dataset, including source identifiers and DOI information, is provided as Dataset S1 in the Supplementary Materials. Additional information supporting the findings of this study is available from the corresponding authors upon reasonable requests.

## Supplementary Materials

The following supporting information can be downloaded at https://www.mdpi.com/article/10.3390/foods15142522/s1, Table S1: Literature search strategy; Table S2: Inclusion and exclusion criteria; Table S3: Data cleaning and record-retention summary; Table S4: Unit harmonization rules; Table S5: Missing-value summary for variables retained in the main modeling dataset; Table S6: Descriptive statistics of continuous variables in the cleaned main modeling dataset; Table S7: Full category distributions of categorical predictors in the cleaned main modeling dataset; Table S8: Summary of five-fold stratified source-grouped validation; Dataset S1: Cleaned main modeling dataset with source identifiers, DOI information, and model-ready variables. The references used for database construction are [2,3,9,10,19,20,21,22,23,24,25,26,27,28,29,30,31,32,33,34].
