# Supplementary material for: Interpretable Machine Learning for Predicting Enrofloxacin Residues in Fish Using a Large Literature-Derived Database
Source: Foods. 2026 Jul 16;15(14):2522. doi: 10.3390/foods15142522 (PMC13408720; doi:10.3390/foods15142522)
Supplement: Supplementary file 1 [file foods-15-02522-s001.zip › foods-4381129-supplementary.pdf]

# Supplementary Materials

## Interpretable Machine Learning for Predicting Enrofloxacin Residues in Fish Using a Large Literature-Derived Database

**Note:** This supplementary file corresponds to the revised main manuscript and summarizes the literature-search strategy, inclusion/exclusion criteria, data cleaning, unit harmonization, missing-value handling, descriptive statistics, full categorical distributions, and source-grouped validation summary. The cleaned modeling dataset contains 1254 records from 39 source groups with traceable literature identifiers. Dataset S1 should be submitted separately as an Excel file containing source identifiers, DOI information, and model-ready variables.

### Table S1. Literature search strategy.

| Database | Search focus | Example search terms | Search date | Article type | Purpose in this study |
|---|---|---|---|---|---|
| Web of Science | Enrofloxacin residue, pharmacokinetics, tissue distribution, and residue depletion in fish | enrofloxacin AND fish AND residue; enrofloxacin AND withdrawal AND fish; enrofloxacin AND tissue distribution | Up to 20 May 2026 | Original articles | Core source for database construction |
| Scopus | Pharmacokinetics, tissue distribution, and residue kinetics in fish | enrofloxacin AND fish AND pharmacokinetics; enrofloxacin AND fish AND residue depletion | Up to 20 May 2026 | Original articles | Cross-database retrieval and source checking |
| PubMed | Veterinary pharmacology and residue-related studies | enrofloxacin AND fish AND pharmacokinetics; enrofloxacin AND aquaculture AND residue | Up to 20 May 2026 | Original articles | Biomedical and veterinary pharmacology source |
| CNKI | Chinese-language literature on aquatic enrofloxacin residues | enrofloxacin AND fish AND residue; enrofloxacin AND aquaculture AND withdrawal period; enrofloxacin AND pharmacokinetics | Up to 20 May 2026 | Original articles | Chinese-language source checking |
| Google Scholar | Supplementary search and citation chasing | enrofloxacin fish residue depletion; enrofloxacin withdrawal time fish; enrofloxacin tissue distribution fish | Up to 20 May 2026 | Original articles | Supplementary retrieval and cross-checking |

**Note:** The search focused on fish-related enrofloxacin residue data suitable for structured extraction. Records outside the fish-related biological-matrix scope were excluded from the final main modeling dataset.

### Table S2. Inclusion and exclusion criteria.

| Category | Inclusion criteria | Exclusion criteria |
|---|---|---|
| Study type | Original experimental studies | Reviews, conference abstracts, comments, and editorials |
| Target drug | Studies reporting parent enrofloxacin concentration | Studies not involving enrofloxacin or only reporting other antibiotics |
| Species scope | Fish-related biological matrices | Non-fish aquatic animals, terrestrial animals, plants, or non-biological environmental samples |
| Matrix type | Fish tissue, plasma, serum, or other fish biological matrices | Water, sediment, feed, plant matrices, environmental-only samples, and method-validation spiked samples |
| Data type | Quantitative enrofloxacin concentration data linked to experimental conditions | Qualitative descriptions only or non-extractable results |
| Extractability | Records linkable to species, matrix, dose, route, sampling time point, or concentration | Records not linkable to a defined observation unit |
| Modeling availability | Records with source information, response value, sampling time point, and positive dose | Records with missing source, missing response, missing sampling time point, or missing/non-positive dose |
| Study relevance | Residue depletion, tissue distribution, pharmacokinetics, residue kinetics, or withdrawal-related studies | Toxicity-only, microbiology-only, environmental occurrence-only, or unrelated analytical-method studies |

## Table S3. Data cleaning and record-retention summary.

| Step | Description | Number retained / flagged | Notes |
|---|---|---|---|
| Initial retrieval | Potentially relevant publications identified | 1128 publications | Retrieved from Web of Science, PubMed, Scopus, CNKI, and Google Scholar. |
| Literature screening | Eligible studies retained for raw extraction | 321 studies | After title, abstract, and full-text screening. |
| Raw extraction | Raw literature-derived records extracted | 2275 | Before duplicate removal and scope filtering. |
| Duplicate checking | Exact duplicate records flagged | 643 | Exact duplicates were identified based on all extracted fields. |
| Duplicate removal | Records retained after exact duplicate removal | 1632 | Intermediate dataset before further scope and critical-variable filtering. |
| Final cleaning | Fish-focused filtering, source cleaning, and exclusion of records with missing critical modeling variables | 1254 | Final main modeling dataset used for model development and validation. |
| Final source grouping | Source groups with traceable literature identifiers | 39 | Source_Group was used for source-grouped validation and provenance tracking, but not as a model predictor. |

## Table S4. Unit harmonization rules.

| Variable | Original reporting forms | Standardized unit | Harmonization rule | Notes |
|---|---|---|---|---|
| Dose | mg/kg, mg kg-1 body weight, or dose reported in treatment description | mg kg-1 body weight | Converted to mg kg-1 body weight where possible | Records with missing or non-positive dose were excluded from the main modeling dataset. |
| Sampling time point | h, day, or min | h | day × 24; min / 60 | Required modeling variable. |
| Water temperature | °C | °C | Retained as reported | Missing values were imputed using the training-set median within each validation scheme. |
| Water pH | pH | unitless pH | Retained as reported | Missing values were imputed using the training-set median within each validation scheme. |
| Fish weight | g or kg | g | kg × 1000 | Missing values were imputed using the training-set median within each validation scheme. |
| Solid fish-tissue concentration | μg/g, mg/kg, ng/g, or source-equivalent tissue units | μg g-1 wet weight | mg/kg = μg/g; ng/g ÷ 1000 | Used as matrix-specific concentration; tissue or biological matrix was retained as a categorical predictor. |
| Plasma, serum, or fluid-matrix concentration | μg/mL, mg/L, ng/mL, or source-equivalent fluid units | μg mL-1 | mg/L = μg/mL; ng/mL ÷ 1000 | Kept as matrix-specific concentration and interpreted together with matrix type. |

## Table S5. Missing-value summary for variables retained in the main modeling dataset.

| Variable | Variable type | Missing records | Missing proportion (%) | Records encoded as Unknown | Handling strategy |
|---|---|---|---|---|---|
| Response variable | Continuous | 0 | 0.00 | 0 | Required response variable; records with missing values were excluded. |
| Species | Categorical | 0 | 0.00 | 0 | Missing or ambiguous entries encoded as Unknown when present. |
| Tissue or biological matrix | Categorical | 0 | 0.00 | 0 | Missing or ambiguous entries encoded as Unknown when present. |
| Administration route | Categorical | 0 | 0.00 | 0 | Missing or ambiguous entries encoded as Unknown when present. |
| Dose | Continuous | 0 | 0.00 | 0 | Required modeling variable; missing or non-positive values were excluded. |
| Sampling time point | Continuous | 0 | 0.00 | 0 | Required modeling variable; missing values were excluded. |
| Water temperature | Continuous | 19 | 1.52 | 0 | Median imputation estimated from the training set within each validation scheme. |
| Water pH | Continuous | 215 | 17.15 | 0 | Median imputation estimated from the training set within each validation scheme. |

| Variable | Variable type | Missing records | Missing proportion (%) | Records encoded as Unknown | Handling strategy |
|---|---|---|---|---|---|
| Fish weight | Continuous | 41 | 3.27 | 0 | Median imputation estimated from the training set within each validation scheme. |
| Health status | Categorical | 0 | 0.00 | 8 | Missing or ambiguous entries encoded as Unknown. |
| Enrofloxacin formulation | Categorical | 0 | 0.00 | 667 | Missing or ambiguous entries encoded as Unknown. |
| Dosing frequency | Categorical | 0 | 0.00 | 1 | Missing or ambiguous entries encoded as Unknown. |

**Note:** Categorical entries explicitly coded as Unknown were retained as informative categories rather than removed. Continuous missing values were imputed only within the model-training pipeline using the training-set median.

## Table S6. Descriptive statistics of continuous variables in the cleaned main modeling dataset.

| Variable | Unit | N | Mean | SD | Median | Q1 | Q3 | Min | Max |
|---|---|---|---|---|---|---|---|---|---|
| Matrix-specific enrofloxacin concentration | matrix-specific concentration | 1254 | 1.984 | 5.090 | 0.280 | 0.001 | 1.595 | 0.000 | 82.80 |
| log1p concentration | log1p scale | 1254 | 0.612 | 0.809 | 0.247 | 0.001 | 0.954 | 0.000 | 4.428 |
| Dose | mg kg-1 body weight | 1254 | 13.76 | 10.57 | 10.00 | 10.00 | 10.00 | 5.000 | 50.00 |
| Sampling time point | h | 1254 | 48.12 | 114.0 | 12.00 | 1.200 | 48.00 | 0.000 | 1416.0 |
| Water temperature | °C | 1235 | 14.88 | 5.914 | 15.00 | 9.700 | 16.30 | 9.700 | 30.20 |
| Water pH | unitless | 1039 | 7.209 | 0.204 | 7.200 | 7.050 | 7.200 | 6.700 | 8.000 |
| Fish weight | g | 1213 | 206.3 | 85.46 | 195.0 | 195.0 | 204.0 | 33.50 | 410.0 |

## Table S7. Full category distributions of categorical predictors in the cleaned main modeling dataset.

| Predictor | Category | Records | Proportion (%) |
|---|---|---|---|
| Species | Salmo salar | 477 | 38.04 |
| Species | Oncorhynchus mykiss | 289 | 23.05 |
| Species | Scophthalmus maximus | 120 | 9.57 |
| Species | Cyprinus carpio haematopterus | 116 | 9.25 |
| Species | Micropterus salmoides | 74 | 5.90 |
| Species | Channa argus | 58 | 4.63 |
| Species | Salmo trutta | 53 | 4.23 |
| Species | Carassius auratus gibelio | 37 | 2.95 |
| Species | Takifugu flavidus | 12 | 0.96 |
| Species | Pangasianodon hypophthalmus | 7 | 0.56 |
| Species | Ctenopharyngodon idella | 4 | 0.32 |
| Species | Korean catfish | 4 | 0.32 |
| Species | Oreochromis niloticus | 3 | 0.24 |
| Tissue or biological matrix | Plasma | 384 | 30.62 |
| Tissue or biological matrix | Liver | 174 | 13.88 |
| Tissue or biological matrix | Kidney | 149 | 11.88 |
| Tissue or biological matrix | Muscle | 131 | 10.45 |
| Tissue or biological matrix | Skin | 97 | 7.74 |
| Tissue or biological matrix | Gill | 85 | 6.78 |
| Tissue or biological matrix | Brain | 68 | 5.42 |
| Tissue or biological matrix | Muscle_skin | 64 | 5.10 |
| Tissue or biological matrix | Gut | 47 | 3.75 |
| Tissue or biological matrix | Bile | 17 | 1.36 |
| Tissue or biological matrix | Plasma_or_serum | 16 | 1.28 |
| Tissue or biological matrix | Bone | 12 | 0.96 |

| Predictor | Category | Records | Proportion (%) |
|---|---|---|---|
| Tissue or biological matrix | Serum | 10 | 0.80 |
| Administration route | Oral | 767 | 61.16 |
| Administration route | Intravenous | 194 | 15.47 |
| Administration route | Bath_or_waterborne | 90 | 7.18 |
| Administration route | Intramuscular | 86 | 6.86 |
| Administration route | Intraperitoneal | 85 | 6.78 |
| Administration route | Medicated_feed | 24 | 1.91 |
| Administration route | Other | 8 | 0.64 |
| Dosing frequency | Single | 1204 | 96.01 |
| Dosing frequency | Repeated | 49 | 3.91 |
| Dosing frequency | Unknown | 1 | 0.08 |
| Enrofloxacin formulation | Unknown | 667 | 53.19 |
| Enrofloxacin formulation | Oral_solution | 263 | 20.97 |
| Enrofloxacin formulation | Solution | 167 | 13.32 |
| Enrofloxacin formulation | Medicated_feed | 68 | 5.42 |
| Enrofloxacin formulation | Enrofloxacin_formulation_unspecified | 47 | 3.75 |
| Enrofloxacin formulation | Other | 42 | 3.35 |
| Health status | Healthy | 1241 | 98.96 |
| Health status | Unknown | 8 | 0.64 |
| Health status | Infected_or_challenge_model | 5 | 0.40 |

## Table S8. Summary of five-fold stratified source-grouped validation.

| Item | Description |
|---|---|
| Grouping variable | Source_Group |
| Number of source groups | 39 |
| Number of modeling records | 1254 |
| Validation strategy | Five-fold stratified source-grouped validation |
| Stratification target | Binned log1p-transformed matrix-specific enrofloxacin concentration |

| Model | $R^2$_log (mean ± SD) | RMSE_log (fold mean) |
|---|---|---|
| ExtraTrees | -0.3409 ± 0.5023 | 0.7978 |
| ElasticNet | -0.5261 ± 0.8683 | 0.8433 |
| RF | -0.9608 ± 1.4324 | 0.9056 |
| TabResNet | -1.3604 ± 1.8396 | 0.9676 |
| SVR | -1.3912 ± 2.0492 | 0.9561 |
| FT-Transformer | -1.5749 ± 2.4346 | 0.9760 |
| MLP | -1.7552 ± 1.5110 | 1.1740 |
| HGBDT | -1.8137 ± 2.6146 | 1.0138 |

**Note:** This table reports the source-grouped validation summary used in the revised main manuscript. If fold-level RMSE_log and MAE_log outputs are exported from the modeling script, they can be appended here or provided as an additional worksheet in Dataset S1. No fold-level values were inferred or fabricated in this supplementary file.

**Supplementary Reference List S1. Source Publications Used in the Literature-Derived Database**

**Note:** Source_Group was used as a provenance-tracking and source-grouped validation identifier rather than as a one-to-one publication identifier. A single publication may contribute multiple Source_Group labels when different species, tissue matrices, administration routes, dosing regimens, or extracted endpoints were used. The following list provides the publication-level bibliographic information for the source studies used in constructing the literature-derived database.


R01. Fang, X.; Liu, X.; Liu, W.; Lu, C. Pharmacokinetics of enrofloxacin in allogynogenetic silver crucian carp, Carassius auratus gibelio. Journal of Veterinary Pharmacology and Therapeutics 2012, 35(4), 397–401. https://doi.org/10.1111/j.1365-2885.2011.01337.x.

R02. Fang, X.; Zhou, J.; Liu, X. Pharmacokinetics of enrofloxacin in snakehead fish, Channa argus. Journal of Veterinary Pharmacology and Therapeutics 2016, 39(2), 209–212. https://doi.org/10.1111/jvp.12262.

R03. Yang, F.; Zhang, C.-S.; Duan, M.-H.; Wang, H.; Song, Z.-W.; Shao, H.-T.; Ma, K.-L.; Yang, F. Pharmacokinetics and Tissue Distribution of Enrofloxacin Following Single Oral Administration in Yellow River Carp (Cyprinus carpio haematopterus). Frontiers in Veterinary Science 2022, 9, 822032. https://doi.org/10.3389/fvets.2022.822032.

R04. Shan, Q.; Wang, J.; Zheng, G.; Zhu, X.; Yang, Y.; Ma, L.; Zhao, C.; Li, L.; Yin, Y. Pharmacokinetics and tissue residues of enrofloxacin in the largemouth bass (Micropterus salmoides) after oral administration. Journal of Veterinary Pharmacology and Therapeutics 2020, 43(2), 147–152. https://doi.org/10.1111/jvp.12794.

R05. Lucchetti, D.; Fabrizi, L.; Guandalini, E.; Podestà, E.; Marvasi, L.; Zaghini, A.; Coni, E. Long Depletion Time of Enrofloxacin in Rainbow Trout (Oncorhynchus mykiss). Antimicrobial Agents and Chemotherapy 2004, 48(10), 3912–3917. https://doi.org/10.1128/AAC.48.10.3912-3917.2004.

R06. Urzúa, N.; Messina, M.J.; Prieto, G.; Lüders, C.; Errecalde, C. Pharmacokinetics and tissue disposition of enrofloxacin in rainbow trout after different routes of administration. Xenobiotica 2020, 50(10), 1236–1241. https://doi.org/10.1080/00498254.2020.1747119.

R07. Bowser, P.R.; Wooster, G.A.; St Leger, J.; Babish, J.G. Pharmacokinetics of enrofloxacin in fingerling rainbow trout (Oncorhynchus mykiss). Journal of Veterinary Pharmacology and Therapeutics 1992, 15(1), 62–71. https://doi.org/10.1111/j.1365-2885.1992.tb00987.x.

R08. Uney, K.; Terzi, E.; Durna Corum, D.; Ozdemir, R.C.; Bilen, S.; Corum, O. Pharmacokinetics and Pharmacokinetic/Pharmacodynamic Integration of Enrofloxacin Following Single Oral Administration of Different Doses in Brown Trout (Salmo trutta). Animals 2021, 11(11), 3086. https://doi.org/10.3390/ani11113086.

R09. Stoffregen, D.A.; Wooster, G.A.; Bustos, P.S.; Bowser, P.R.; Babish, J.G. Multiple route and dose pharmacokinetics of enrofloxacin in juvenile Atlantic salmon. Journal of Veterinary Pharmacology and Therapeutics 1997, 20(2), 111–123. https://doi.org/10.1046/j.1365-2885.1997.81531.x.

R10. Ma, R.; Yang, L.; Ren, T.; Dong, Y.; Liu, T.; Zhuang, P.; Fang, W.; Yang, X.; Hu, K. Enrofloxacin pharmacokinetics in Takifugu flavidus after oral administration at three salinity levels. Aquaculture Research 2017, 48(8), 4545–4553. https://doi.org/10.1111/are.13279.

R11. Xu, L.; Wang, H.; Yang, X.; Lu, L. Integrated pharmacokinetics/pharmacodynamics parameters-based dosing guidelines of enrofloxacin in grass carp Ctenopharyngodon idella to minimize selection of drug resistance. BMC Veterinary Research 2013, 9, 126. https://doi.org/10.1186/1746-6148-9-126.

R12. Shan, Q.; Wang, J.X.; Wang, J.; Ma, L.S.; Yang, F.H.; Yin, Y.; Huang, R.; Liu, S.G.; Li, L.C.; Zheng, G.M. Pharmacokinetic/pharmacodynamic relationship of enrofloxacin against Aeromonas hydrophila in crucian carp (Carassius auratus gibelio). Journal of Veterinary Pharmacology and Therapeutics 2018, 41(6), 887–893. https://doi.org/10.1111/jvp.12678.

R13. Fan, J.; Shan, Q.; Wang, J.; Liu, S.; Li, L.; Zheng, G. Comparative pharmacokinetics of enrofloxacin in healthy and Aeromonas hydrophila-infected crucian carp (Carassius auratus gibelio). Journal of Veterinary Pharmacology and Therapeutics 2017, 40(5), 580–582. https://doi.org/10.1111/jvp.12392.

R14. Shan, Q.; Fan, J.; Wang, J.; Zhu, X.; Yin, Y.; Zheng, G. Pharmacokinetics of enrofloxacin after oral, intramuscular and bath administration in crucian carp (Carassius auratus gibelio). Journal of Veterinary Pharmacology and Therapeutics 2018, 41(1), 159–162. https://doi.org/10.1111/jvp.12428.

R15. Shan, Q.; Huang, H.; Zheng, G.; Yin, Y.; Zhu, X.; Ma, L.; Zhou, H.; Xie, W.; Li, L.; Liu, S.; Wang, J. Pharmacokinetics and Tissue Residue Profiles of Enrofloxacin in Crucian Carp (Carassius auratus gibelio) Following Single and Multiple Oral Administration. Frontiers in Veterinary Science 2022, 9, 872828. https://doi.org/10.3389/fvets.2022.872828.

R16. Kim, M.-S.; Lim, J.-H.; Park, B.-K.; Hwang, Y.-H.; Yun, H.-I. Pharmacokinetics of enrofloxacin in Korean catfish (Silurus asotus). Journal of Veterinary Pharmacology and Therapeutics 2006, 29(5), 397–402. https://doi.org/10.1111/j.1365-2885.2006.00783.x.

R17. Zhang, W.; Wang, J.; Zheng, G.; Yin, Y.; Zhu, X.; Shan, Q.; Yang, Y.; Ma, L.; Li, L.; Liu, S. Pharmacokinetics, tissue distribution, and depletion of enrofloxacin and its metabolite ciprofloxacin in the northern snakehead (Channa argus) following multiple oral administration. Aquaculture 2021, 533, 736183. https://doi.org/10.1016/j.aquaculture.2020.736183.

R18. Liang, J.P.; Li, J.; Zhao, F.Z.; Liu, P.; Chang, Z.Q. Pharmacokinetics and tissue behavior of enrofloxacin and its metabolite ciprofloxacin in turbot Scophthalmus maximus at two water temperatures. Chinese Journal of Oceanology and Limnology 2012, 30(4), 644–653. https://doi.org/10.1007/s00343-012-1228-2.

R19. Xu, W.; Zhu, X.; Wang, X.; Deng, L.; Zhang, G. Residues of enrofloxacin, furazolidone and their metabolites in Nile tilapia (Oreochromis niloticus). Aquaculture 2006, 254(1–4), 1–8. https://doi.org/10.1016/j.aquaculture.2005.10.030.

R20. Phu, T.M.; Douny, C.; Scippo, M.-L.; De Pauw, E.; Nguyen, Q.T.; Huong, D.T.T.; Vinh, H.P.; Nguyen Thanh, P.; Dalsgaard, A. Elimination of enrofloxacin in striped catfish (Pangasianodon hypophthalmus) following on-farm treatment. Aquaculture 2015, 438, 1–5. https://doi.org/10.1016/j.aquaculture.2014.12.032.

**Dataset S1. Cleaned main modeling dataset with source identifiers, DOI information, and model-ready variables.**

**Note:** Dataset S1 should be submitted as a separate Excel file. At minimum, it should retain Source_Group, Literature_ID_clean, DOI or DOI-containing source information, Observation_Unit_ID, Curve_ID, all 11 model predictors, matrix-specific enrofloxacin concentration, and the model-use indicators.
